# Benefit of Bi-Ocular Visual Stimulation for Postural Control in Children with Strabismus

**DOI:** 10.1371/journal.pone.0060341

**Published:** 2013-04-02

**Authors:** Chrystal Gaertner, Charlotte Creux, Marie-Andrée Espinasse-Berrod, Christophe Orssaud, Jean-Louis Dufier, Zoï Kapoula

**Affiliations:** 1 IRIS Group, Centre d’Etudes SensoriMotrices UMR8194, CNRS, Université Paris Descartes, Paris, France; 2 Service d’Ophtalmologie, Hôpital pour enfants Necker, Paris, France; University of Rome, Italy

## Abstract

Vision is important for postural control as is shown by the Romberg quotient (RQ): with eyes closed, postural instability increases relative to eyes open (RQ = 2). Yet while fixating at far distance, postural stability is similar with eyes open and eyes closed (RQ = 1). Postural stability can be better with both eyes viewing than one eye, but such effect is not consistent among healthy subjects. The first goal of the study is to test the RQ as a function of distance for children with convergent versus divergent strabismus. The second goal is to test whether vision from two eyes relative to vision from one eye provides better postural stability. Thirteen children with divergent strabismus and eleven with convergent strabismus participated in this study. Posturtography was done with the Techno concept device. Experiment 1, four conditions: fixation at 40 cm and at 200 cm both with eyes open and eyes covered (evaluation of RQ). Experiment 2, six conditions: fixation at 40 cm and at 200 cm, with both eyes viewing or under monocular vision (dominant and non-dominant eye). For convergent strabismus, the groups mean value of RQ was 1.3 at near and 0.94 at far distance; for divergent, it was 1.06 at near and 1.68 at far. For all children, the surface of body sway was significantly smaller under both eyes viewing than monocular viewing (either eye). Increased RQ value at near for convergent and at far for divergent strabismus is attributed to the influence of the default strabismus angle and to better use of ocular motor signals. Vision with the two eyes improves postural control for both viewing distances and for both types of strabismus. Such benefit can be due to complementary mechanisms: larger visual field, better quality of fixation and vergence angle due to the use of visual inputs from both eyes.

## Introduction

Postural control is based on an integration of information of different inputs: visual, proprioceptive, somesthesic and vestibular. The importance of vision is studied via the Romberg test: numerous studies compared postural stability of adults with eyes open versus closed [1], [2], [3]; in such a test the surface of body sway was reported to be 2 to 3 times more with eyes closed than with eyes open.

Postural stability depends also on the distance at which the subject is fixating; it is better when fixating a target at near distance than at far: retinal slip induced by a body sway is more pronounced at near distance than at far, thus triggering more corrective action on posture [4]. In addition, the vergence angle is also more important in near vision. Kapoula and Lê [5] demonstrated that the increase of ocular convergence via prisms alone is sufficient to improve instantaneously postural stability. They attributed this effect to the efferent and afferent information resulting from the high degree of convergence of the eyes at a near distance.

Lê and Kapoula [6] showed that the Romberg Quotient (RQ), which is the ratio eye closed/eye open, also depends on viewing distance: when tested at near distance, the RQ is about 2 (i.e., stability with eyes closed is indeed about 2 times worse than with eyes open), but at a far distance the RQ was found to drop to 1. In a complementary study, the authors recorded eye movements and measured the change in vergence angle during the Romberg test, comparing room light and room dark conditions; they showed that the increased value of the RQ at near distance was closely correlated with the large difference in the convergence angle between light and dark condition, as it was not possible to maintain the eyes converged in the dark for more than a few seconds. Thus, vergence difference between light and dark being higher at near distance than at far, this translates to higher postural difference between the dark-light conditions and thus to higher RQ values at near. Lê and Kapoula [6] concluded that the physiological meaning of the RQ should be extended as it indicates both the importance of vision and of the oculomotor signals related to vergence.

Another important aspect of postural control in a quiet stance is whether binocular vision leads to better stability than monocular vision. Evidence for a superiority of binocular vision is rather weak: binocular vision was found to be profitable only for a small group of healthy adult subjects, e.g., leading to a better stability [7], [8]. Gentaz [9] advanced the hypothesis that there is a preferred eye, called the "postural eye", not necessarily the dominant eye, which is used preferentially to maintain best postural stability. Lê and Kapoula [10] provided further evidence for moderate superiority of binocular vision: they reported that the proximity benefit (e.g., better stability while fixating at near than at far) exists only for binocular vision.

This study examines the RQ as a function of viewing distance and the binocular-monocular viewing question in a population of children with convergent and divergent strabismus. Strabismus affects about 3 to 4% of children in the 6 first years of life. For children with early onset strabismus occurring (i.e., the first two years of life) binocular vision is absent and diplopia may exist. Some studies found that strabismus adult patients have a reduced capacity to keep their body stable, compared to controls [11], [12], but to our knowledge, no studies explored the Romberg Quotient, neither the importance of binocular versus monocular viewing in convergent versus divergent strabismic children. A cases study report from Legrand et al. [13] showed that strabismus children are more stable with open eyes than closed eyes both at near and at far distances: in this study, only 9 children were studied, 3 of them had exotropia. Thus there is a need for further research in this field, to investigate the importance of vision per se; its eventual interaction with viewing distance and the importance of binocular versus monocular vision at far or at near. In this study, we will use the term bi-ocular since the majority of the children with strabismus we studied have no single binocular foveal vision; the term bi-ocular describes the presence of visual stimulation of the two eyes, and perhaps gross peripheral binocular vision.

This study focuses on the comparison of convergent versus divergent strabismus and does not include a control group. Yet, some comparison will be made in the Discussion with the control data from another study [14] using similar testing conditions in children of a similar age (11 years old).

## Materials and Methods

### Subjects

For the two experiments, twenty four strabismic children participated at this study (mean age 11.25±3.69 years), eleven of whom presented with convergent strabismus and thirteen of whom presented with divergent strabismus. They were recruited at Necker Hospital's ophthalmology service during their regular follow-up visit. It should be noted that those children received regular follow-ups at the ophthalmology service since their early childhood in order to take prophylactic measures against amblyopia. So, all demonstrated good visual acuity (>8/10 for each eye).

The investigation adhered to the principles of the Declaration of Helsinki and was approved by our institutional human experimentation committee, the “Comité de Protection des Personnes” (CPP) Ile de France VI (No: 07035), Necker Hospital in Paris, France. Written, informed parental consent was obtained from the participant's parents after the nature of the experimentation had been explained.

### Clinical characteristics

Table 1 shows the clinical characteristics for each subject. Thirteen children underwent strabismus surgery, two of them for the second time (S16, S26). All children were tested at least one month after surgery, when all children had adapted to the new ocular deviation and the strabismus angle was relatively stable. Among the post-operative cases, three of the subjects differed in the type of strabismic condition presented before and after the surgery: S3 and S11 had convergent strabismus before and divergent strabismus after surgery, and S31 had divergent strabismus before and convergent strabismus after surgery. Also, one subject (S5) had preoperative convergent strabismus and postoperative esotropia upon fixation at far and exotropia upon fixation at near distances. The ten other subjects had the same type of strabismus before and after surgery, five convergent (S6, S14, S21, S26 and S33) and four divergent (S9, S16, S28 and S29). In the children that underwent surgery, five presented some stereo acuity (S14, S16, S28, S29, and S33) measured with the TNO test. Eleven children underwent no surgery (S1, S4, S10, S12, S13, S15, S18, S22, S24, S30 and S32); seven of them had some stereo acuity (S1, S4, S10, S13, S18, S22 and S30), and six of them had divergent intermittent strabismus.

**Table 1:** Clinical characteristics of children with strabismus.

Subjects*	Age (year)	Visual acuity	Dominant eye	angle of strabismus (prism D)	stéréoacuity	surgery
1	17	RE 8/10 LE 4/10	RE	XT 35 far IXT 40 near	400″	pre
2	16	RE 10/10 LE 8/10	RE	XT 2 far XT 10 near		post
3	7	RE 10/10 LE 10/10	LE	XT 35 far IXT 16 near	40″	pre
4	13	RE 10/10 LE 8/10	RE	ET 6 far XT4 near		post
5	10	RE 10/10 LE 5/10	RE	ET 25 far ET 20 near		post
6	15	RE 10/10 LE 10/10	RE	XT 2 far XT 2 near		post
7	12	RE 10/10 LE 10/10	LE	IXT 45 far IXT 50 near	240″	pre
8	15	RE 10/10 LE 10/10	LE	XT 8 far XT 10 near		post
9	7	RE 9/10 LE 10/10	LE	XT 10 far XT 4 near		pre
10	9	RE 9/10 LE 9/10	RE	XT 50 far IXT 35 near	40″	pre
11	17	RE 10/10 LE 10/10	RE	E 10 far E 12 near	200″	post
12	7	RE 10/10 LE 10/10	LE	ET 65 far ET 65 near		pre
13	11	RE 10/10 LE10/10	RE	XT 18 far IXT 10–20 near	40″	post (2)
14	7	RE 10/10 LE 8/10	RE	XT 20 far IXT 45–50 near	100″	pre
15	16	RE 10/10 LE 10/10	RE	ET 8 far ET 14 near		post
16	7	RE 8–9/10 LE 9/10	RE	ET 35 far ET 55 near	100″	pre
17	6	RE 10/10 LE 10/10	RE	ET 45 far ET 55 near		pre
18	10	RE 10/10 LE 9/10	RE	ET14 far ET 18 near		post (2)
19	10	RE 10/10 LE 10/10	RE	IXT 4 far IXT 6 near	40″	post
20	10	RE 10/10 LE 10/10	RE	IXT 14 far IXT 16 near	40″	post
21	12	RE 10/10 LE 10/10		X16 far IXT 40 near	40″	pre
22	16	RE 10/10 LE 10/10	RE	ET 2 far ET 4 near		post
23	13	RE 10/10 LE 9/10	RE	ET 10 far ET 35 near		pre
24	8	RE 9/10 LE 10/10	LE	ET 1 far ET 4 near	200″	post

RE: right eye, LE: left eye, All subjects had a good visual acuity in both eyes. Stereoacuity was assessed with the TNO test (no values means that there was no measurable stereoacuity). Type of deviation: XT, exotropia; IXT, intermittent exotropia; ET, esotropia.

For all but two children (S9 and S15) deviation depended on viewing distance. All subjects had good visual acuity in both eyes, except subject 1, measured with Parinaud test for near vision (33 cm) or with the Pigassou drawing test for young children who did not read; for far vision (5 m), the Monoyer scale was used.

During the experiment, the subjects wore their habitual corrective lenses. The dominant eye was the fixating eye, measured with the unilateral cover-test: the subject fixated a target at 5 m and the orthoptist covered alternately each eye observing the viewing eye. If the viewing eye did not move, then it was considered as the dominant, fixating eye. This test is one of most commonly used techniques in clinical evaluation.

### Posturography

We recorded the postural stability with a Feetest 1 device that has two balance footpads (principle of stain gauge) produced by Technoconcept (Céreste, France). The excursions of the center of pressure (CoP) were measured during 25.6 seconds; the equipment contained an analog to digital converter of 16 bits. The sampling frequency of the CoP was 40 Hz.

### Procedure

The children were required to stand upright on the force platform, barefoot (arms side by side, normal breathing, not speaking and with teeth not clenched) feet placed side by side at an angle of 30° and with their heels separated by 4 cm.

The first experiment run was the Romberg Quotient test (RQ) by measuring posture in eyes open and eyes covered conditions: subjects were asked to fixate a target cross placed at eye level at near distance (40 cm, requiring a vergence angle of 9.23°), then the eyes were covered with a sleep band and subjects were asked to continue fixating the imagined target. For far distance, subjects fixated a target also placed at eye level at 200 cm (requiring a vergence angle of 1.86°), then the eyes were also covered with a sleep eye band and subjects were asked to continue fixate at the imagined previously fixated target. The order of the condition far versus near viewing was counterbalanced.

In the second experiment, children had to fixate a target placed at eye level either with both eyes viewing (BEV) or with one eye covered: the dominant eye (DE), or the non-dominant eye (NDE). Those three conditions were repeated at far (200 cm) and at near viewing distance (40 cm). As for the first experiment, the order of viewing distance was counter balanced; the order of binocular and monocular conditions was also counter balanced. For both experiments, the sleep eye band was placed over the child's head from the beginning of the experiment. A short rest period of at least 2–3 minutes was adopted between any two conditions of the same experiment and a longer time rest between the two experiments (10 minutes).

### Postural parameters

Three postural parameters were used in this study to measure the postural stability: the standard deviation of the medio-lateral body sway (SdX in mm), the standard deviation of the antero-posterior body sway (SdY in mm) and the surface of the Center of Pressure, CoP (size of the Surface area of CoP that contains 90% of closest CoP positions from the central ones, in mm^2^s); the variance of speed of the CoP excursions was also measured. The medio-lateral and antero-posterior body sway are believed to be related to different muscular strategies [15]: the SdX is controlled by a hip strategy while the SdY is controlled by an ankle strategy. As for the variance of speed, it is believed to reflect the energy needed to keep the body stable [16]. For each of these parameters (Surface, SdX, SdY and variance of speed) we calculated the ratio of values obtained for the eyes covered/eyes open conditions. This provided four values of RQ for each viewing distance, i.e. a total of eight values.

### Statistical analysis

For experiment 1 an analysis of multivariate variance (MANOVA) was done on RQ values with independent factors being distance (near versus far) and group (convergent versus divergent). The dependant factors was the two postural parameters (body sway in Medio-lateral and Anteroposterior axis). Two ANOVA analysis were performed on RQ values on the surface and speed variance parameter, with distance (near versus far) being the main factor and the type of Strabismus (convergent or divergent) being the inter-subject factor.

For experiment 2, a repeated measure ANOVA was run with two main factors: the viewing condition (both eyes viewing, dominant eye and non-dominant eye viewing), the distance (far versus near) as the co-variant variable, and the type of strabismus (convergent or divergent) as the inter-group factor.

The post-hoc comparison was done with the Fisher's Least Significant Difference (LSD) test; post-hoc comparison were made only when the ANOVA results was significant (i.e. *p*<0.05).

Kolmogorov-Smirnov tests for data normality revealed a normal distribution in each testing condition. We present here the only parameter for which there was a significant effect.

## Results

### Experiment 1 - Romberg quotient-distance and type of strabismus

Table 2 shows the group means RQ together with the standard error for the four parameters: Surface of CoP, SdX, SdY, and Variance of speed. Results are shown for each viewing distance and for convergent and divergent strabismus. Recall RQ is the ratio obtained in the eyes closed over eyes open conditions.

**Table 2:** Postural stability measurements in quiet stance for experiment 1.

RQ NEAR	SdX	SdY	Surface	Variance of speed
Convergent strabismus				
Mean	1,30	1,23	1,77	1,69
Standard error	0,43	0,56	0,99	0,79
Divergent strabismus				
Mean	1,06	1,36	1,77	1,63
Standard error	0,47	0,41	1,02	0,85

RQ FAR	SdX	SdY	Surface	Variance of speed
Convergent strabismus				
Mean	0,94	1,14	1,29	1,37
Standard error	0,24	0,29	0,49	0,89
Divergent strabismus				
Mean	1,68	1,21	1,80	1,29
Standard error	1,41	0,32	1,42	0,33

Means and standard errors of standard deviations of lateral (SdX) and of anteroposterior (SdY) body sway, surface of CoP, and variance of speed for each conditions i.e., fixation at near (40 cm) with open eyes and with covered eyes and fixation at far (200 cm) with open eyes and with covered eyes and Romberg Quotient at near (40 cm) and at far (200 cm) distance for convergent and divergent strabismus children.

**Figure 1:**
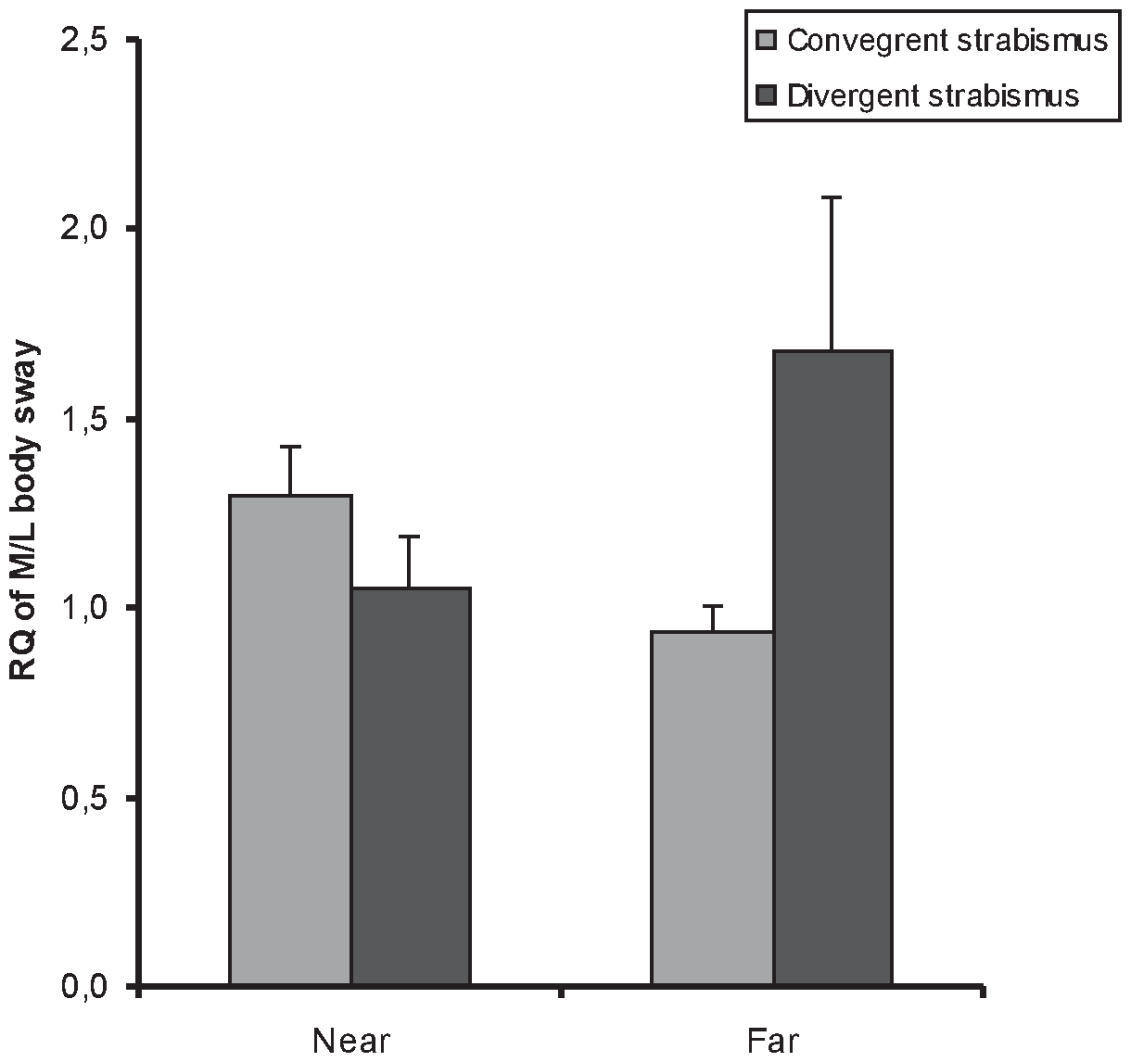
Romberg quotient at near and at far in terms of strabismus. Means of Romberg quotient at near (40 cm) and at far (200 cm) distance for the standard deviation of lateral (SdX) body sway parameter for both convergent and divergent strabismic adolescents. Error bars represent the standard error. Asterisks indicate significant differences (p<0.05).

The MANOVA showed no significant effect of viewing distance condition on the Romberg Quotient (F_(1,42)_ = 0.002, p = 0.96), no effect of the type of strabismus (F_(1,42)_ = 1.61, p = 0.21) and no effect of the parameter (F_(1,42)_ = 0.007, p = 0.93) but there was an interaction between the distance, the type of strabismus and the parameter (F_(1,21)_ = 4.19, p = 0.04). The LSD post-hoc test showed that the RQ is significantly higher for children with divergent strabismus than for children with convergent strabismus at far distance in medio-lateral axis (p = 0.0056). It also showed that the RQ is higher at far distance than at near distance for children with divergent strabismus, also in medio-lateral axis (p = 0.016) (See Figure 1). At near distance, the RQ is about 1.06 ± 0.47 for divergent strabismus, but 1.3 ± 0.43 for convergent strabismus. In contrast, for far distance the RQ is 1.68 ± 1.41 for divergent strabismus and 0.94 ± 0.24 for convergent strabismus.

The two ANOVA tests which were carried out on surface and speed variance showed no effects whatsoever (p>0.05).

### Experiment 2-Viewing condition: bi-ocular versus monocular

Table 3 shows the group mean values for posturography parameters (Surface of CoP, SdX, SdY, Variance of speed) under both eye viewing, dominant eye and non dominant eye viewing conditions for the two types of strabismus (convergent and divergent) and for the two distances (far and near).

**Table 3:** Postural stability measurements in quiet stance for experiment 2.

	NEAR				FAR			
**BEV**	SdX	SdY	Surface	Variance of speed	SdX	SdY	Surface	Variance of speed
Convergent strabismus								
Mean	2,70	4,41	152	136	3,86	5,43	217	195
Standard error	1,32	2,50	114	209	1,55	2,94	127	212
Divergent strabismus								
Mean	3,97	5,32	210	136	3,24	4,72	237	165
Standard error	2,08	2,14	130	145	1,92	1,57	186	183
**DE**	SdX	SdY	Surface	Variance of speed	SdX	SdY	Surface	Variance of speed
Convergent strabismus								
Mean	3,06	5,21	248	81	3,61	5,84	291	159
Standard error	1,57	2,94	299	51	1,15	3,12	169	185
Divergent strabismus								
Mean	3,48	5,30	242	110	4,33	6,77	386	151
Standard error	1,40	2,22	192	82	2,29	2,10	267	162
**NDE**	SdX	SdY	Surface	Variance of speed	SdX	SdY	Surface	Variance of speed
Convergent strabismus								
Mean	3,88	5,55	273	186	4,05	4,23	246	153
Standard error	1,19	2,84	166	219	1,97	1,56	179	181
Divergent strabismus								
Mean	3,89	5,51	323	172	3,90	5,78	296	156
Standard error	2,08	4,11	341	212	2,14	1,81	181	115

Means and standard errors of standard deviations of lateral (SdX) and of anteroposterior (SdY) body sway, surface of CoP, and variance of speed for each conditions i.e., both eyes viewing (BEV), monocular fixation with the dominant eye (DE) and monocular fixation with the non dominant eye (NDE) for convergent and divergent strabismus children.

**Figure 2:**
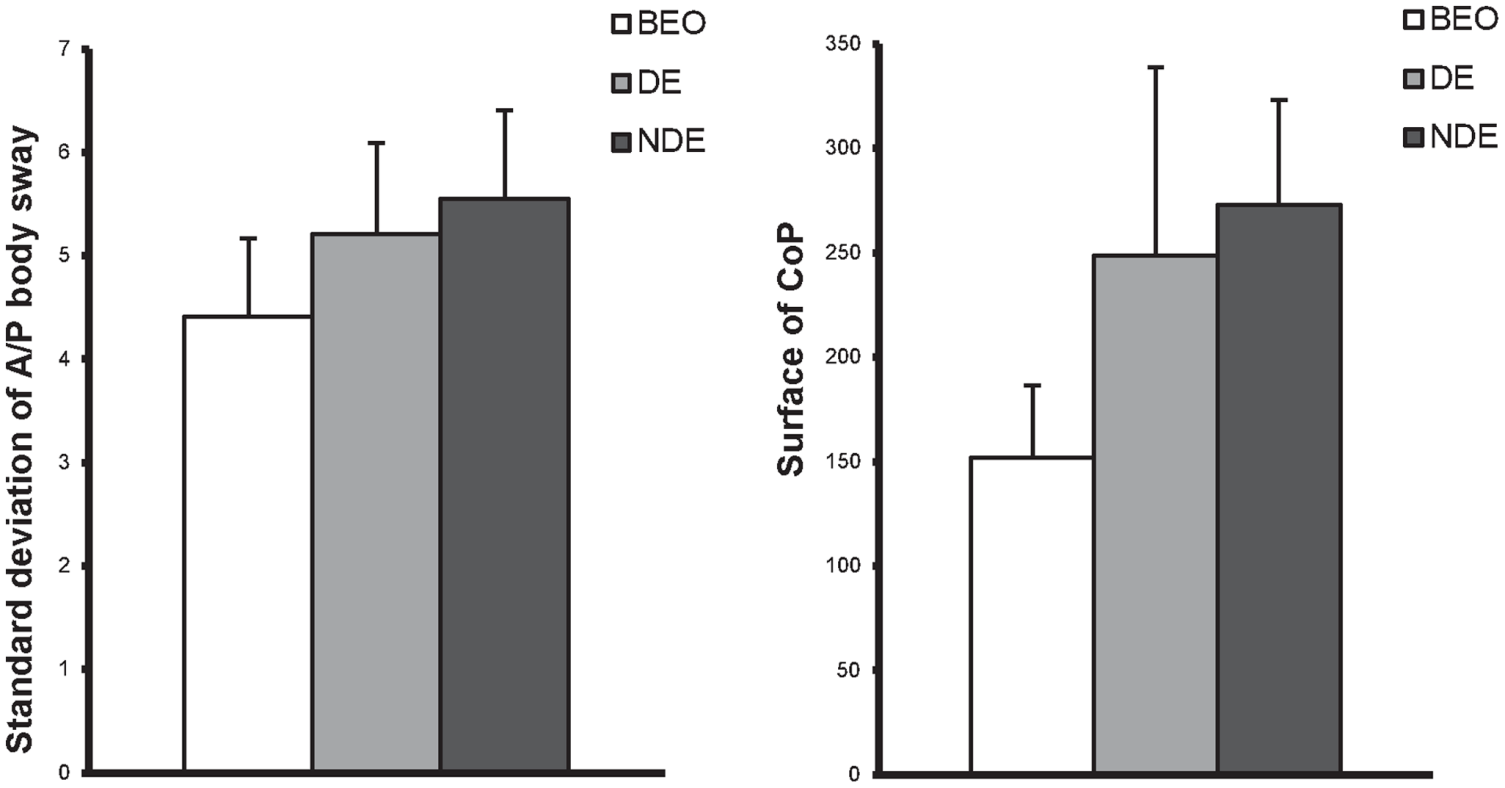
Effect of the viewing condition on postural parameters. Means values of the viewing conditions for the antero-posterior body sway (**A**) and for the Surface of CoP (**B**) for all strabismus children at both distances. Error bars represent the standard error. Asterisks indicate significant differences (p <0.05).

There was a main effect of the viewing condition for the parameters SdY (F_(2,42)_ = 5.6, p = 0.0069). The post-hoc test showed that the antero-posterior body sway was higher under monocular viewing with the dominant eye, comparing to monocular viewing with the non-dominant eye (p = 0.045) or compared with the both eyes viewing condition (p = 0.0017) (See Figure 2A).

There was also an effect of the viewing condition on the surface of the CoP (F_(2,42)_ = 6.27, p = 0.0041). The LSD post-hoc test showed that the surface was smaller under both eyes viewing condition than under either monocular viewing condition, dominant eye (p = 0.0017) or non-dominant eye (p = 0.045) (See Figure 2B).

## Discussion

The main findings of this study are the following: (i) there is an effect of distance on RQ but only for the medio-lateral body sway; for convergent strabismus RQ increases at near distance relative to far, similarly to what has been reported previously for healthy subjects; for divergent strabismus the opposite pattern is observed, RQ is higher at far than at near distance; (ii) viewing with both eyes produces better postural stability in strabismus children, regardless of the type of strabismus and of the viewing distance.

### Romberg Quotient distance dependency

Children with convergent strabismus showed higher RQ value at near vision (1.3) than at far (0.94). In other words, difference in postural stability, in terms of medio-lateral body sway between eyes open and eyes covered condition was higher at near distance than at far distance. As mentioned, a similar effect was observed in healthy adults by Lê and Kapoula [6]. Moreover, the study from Bucci et al. [11] that included data from healthy children of average age 11 years, reported similar distance effect in children: from Fig. 1 of this study it can be extrapolated that the RQ value for control children was 1.62 for near distance (40 cm) and 1.30 for far distance (200 cm) for the medio-lateral body sway. Thus, the behavior of children with convergent strabismus in the present study is similar to that of control children as well. In contrast children with divergent strabismus show a different pattern, as their RQ is smaller at near distance (1.06) than at far distance (1.68).

The interpretation we propose is the following: strabismic children use ocular motor and proprioceptive signals (efferent or afferent, related to the angle of vergence) only when the strabismus angle of vergence is grossly in correspondence with the fixation requirement, i.e. for convergent strabismus near fixation, for divergent strabismus far fixation. When both eyes are viewing, such ocular motor signals are used and improve postural stability relative to the eyes covered condition. The difference between eyes open and eye covered conditions is thus higher for near than far distance for convergent strabismus, and for far than near distance for divergent strabismus, leading to higher RQ values.

This interpretation in terms of use or not of oculomotor signals for postural control is in line with that proposed for healthy subjects; the difference for strabismus is that signals are used selectively only when the angle is grossly appropriate for what is required for fixation. Further studies with a gradient of different distances including distances at which the strabismus angle is perfectly adjusted to the fixation requirement would be of interest. Due to time limitations in the availability of children in the hospital such extensive testing was not possible in the present study. It is important to note that these effects are subtle and extremely specific since the difference between convergent and divergent strabismus is only relevant for lateral body sway which is believed to reflect a hip control strategy [16].

In summary, the interpretation of the data is based on the assumption that children with convergent strabismus use convergence signals and children with divergent strabismus use divergence signals in postural control, only when such signals are grossly appropriate relative to the fixation requirement. One could argue that higher RQ values at near is also related to higher angular size of retinal slip that could result from translational lateral body sway. It is known that the gain of translational VOR is higher at near distance [17], [18]. This cannot be excluded, but its validity in strabismus seems to be limited only to subjects with convergent strabismus.

Postural control is a complex multisensory function and the idea of different priority and weighting of different signals is widely accepted (see for a review [19]). This study adds knowledge to this field concerning strabismus.

### Superiority of binocular vision for postural control

This rather unexpected result is of major importance. All children with strabismus, whatever the type (convergent or divergent) are more stable when tested under both eyes viewing condition than under monocular viewing, with the dominant or non dominant eye. The surface of the CoP and the A/P body sway were found to be smaller with both eyes viewing, regardless of the viewing distance. It is surprising because several studies [7], [8], [10] failed to show in control adults such clear superiority of binocular viewing for postural stability. In fact, these authors showed that control adults were as stable in binocular viewing as in monocular viewing and some subjects show even better postural stability under monocular viewing with the dominant eye. So it seems that strabismus children, even though they do not have single binocular vision, control better their posture when both eyes are viewing. We think that both sensory and motor mechanisms are involved. Bucci et al. [11] in their control group of 11 years old children, showed better postural stability for binocular viewing than monocular viewing but only at near distance. The results on strabismus children are partially in line with their study as they show that the benefit of binocular viewing in strabismus children is generalized for both near and far distance. Assaiante and Amblard [20] showed that 11 years old children are more visuo-dependant than older children or adults. Perhaps our results on the importance of bi-ocular viewing can be seen as evidence for bi-ocular visual dependency. Other interpretations will be discussed below.

A first plausible interpretation is related to active vergence angle control involved more or less (according to distance) when both eyes are viewing. Active vergence is controlled better when visual input from the two eyes is available. This is because gross binocular disparity cues in the visual periphery can be used when both eyes are viewing. The angle of strabismus itself is known to vary with eye dominance even when it is considered clinically as constant. There is clinical evidence for large variation of strabismus between binocular and monocular conditions, or with attention and eye dominance [21]. Moreover, under monocular viewing it is possible that some subclinical latent nystagmus occurs that might interfere with postural control. Indeed, Brandt [22] reported a case with specifically increased postural sway in gaze positions where nystagmus was increased. Latent nystagmus is frequent in children with history of early onset strabismus [23].

Further evidence for a link between oculomotor signals and posture comes from studies dealing with eye position and neck muscle activity. Indeed, André-Dushays et al. [24], [25] found both a tonic and a dynamic coupling of the neck splenius muscles with horizontal eye position, in head fixed subjects. They suggest that oculomotor commands can be transmitted to both extra ocular and neck muscles. Furthermore, Han and Lennerstrandt [26] demonstrated that vibration of neck muscles used for lateral head rotation improved the dynamics of accommodative vergence. Thus, the benefit of both eyes viewing condition compared to monocular viewing could come from better eye movement signals e.g. from more stable eye fixation and vergence angle, and from the link between the oculomotor signals and the neck muscle activation all contributing to better postural control and stability. There is definitively need for further studies in this field combining postural measures, eye movements recording and EMG measures.

Finally, a complementary sensory factor is the size of visual field. According to the peripheral dominant hypothesis, peripheral vision cues are most important for the control of posture and self-motion [27], [28], [29], [30]. With both eyes open, the field being larger, one would be able to use better peripheral visual cues to control posture.

It should be emphasized that the benefit for both eyes viewing exists for all children, regardless of the type of strabismus and for both far and near distances. Thus, for the strabismus population, visual stimulation from both eyes, even without single binocular fusion, is a key factor for better postural stability. Such observations highlight the importance of rudimentary bi-ocular vision even in the absence of single fused vision.
